# A human monoclonal antibody blocking SARS-CoV-2 infection

**DOI:** 10.1038/s41467-020-16256-y

**Published:** 2020-05-04

**Authors:** Chunyan Wang, Wentao Li, Dubravka Drabek, Nisreen M. A. Okba, Rien van Haperen, Albert D. M. E. Osterhaus, Frank J. M. van Kuppeveld, Bart L. Haagmans, Frank Grosveld, Berend-Jan Bosch

**Affiliations:** 1 Virology Section, Infectious Diseases and Immunology Division, Department of Biomolecular Health Sciences, Faculty of Veterinary Medicine, Utrecht University, Utrecht, the Netherlands; 2 Department of Cell Biology, Erasmus Medical Center, Rotterdam, the Netherlands; 3 Harbour BioMed, Rotterdam, the Netherlands; 4 Department of Viroscience, Erasmus Medical Center, Rotterdam, the Netherlands; 5 University of Veterinary Medicine, Hannover, Germany

**Keywords:** Immunotherapy, Virology

## Abstract

The emergence of the novel human coronavirus SARS-CoV-2 in Wuhan, China has caused a worldwide epidemic of respiratory disease (COVID-19). Vaccines and targeted therapeutics for treatment of this disease are currently lacking. Here we report a human monoclonal antibody that neutralizes SARS-CoV-2 (and SARS-CoV) in cell culture. This cross-neutralizing antibody targets a communal epitope on these viruses and may offer potential for prevention and treatment of COVID-19.

## Abstract (web summary)

Vaccines and targeted therapeutics for severe acute respiratory syndrome coronavirus 2 (SARS-CoV-2) are currently lacking. Here, the authors report a human monoclonal antibody capable of neutralizing both authentic SARS-CoV and SARS-CoV-2 by targeting a common epitope.

## Introduction

The severe acute respiratory syndrome coronavirus 2 (SARS-CoV-2) is the etiological agent of the coronavirus induced disease 19 (COVID-19) that emerged in China late 2019 and causing a pandemic^1^. As of 19 April 2020, 2,241,778 cases have been reported worldwide, of which 152,551 (6.8%) succumbed to the infection^2^. SARS-CoV-2 belongs to the *Sarbecovirus* subgenus (genus *Betacoronavirus*, family *Coronaviridae*)^3^ together with SARS-CoV that emerged in 2002 causing ~8000 infections with a lethality of 10%. Both viruses crossed species barriers from an animal reservoir and can cause a life-threatening respiratory illness in humans. Presently, no approved targeted therapeutics are available for COVID-19. Monoclonal antibodies targeting vulnerable sites on viral surface proteins are increasingly recognized as a promising class of drugs against infectious diseases and have shown therapeutic efficacy for a number of viruses^4,5^.

Coronavirus-neutralizing antibodies primarily target the trimeric spike (S) glycoproteins on the viral surface that mediate entry into host cells. The S protein has two functional subunits that mediate cell attachment (the S1 subunit, existing of four core domains S1_A_ through S1_D_) and fusion of the viral and cellular membrane (the S2 subunit). Potent neutralizing antibodies often target the receptor interaction site in S1, disabling receptor interactions^6–11^. The spike proteins of SARS-CoV-2 (SARS2-S; 1273 residues, strain Wuhan-Hu-1) and SARS-CoV (SARS-S, 1255 residues, strain Urbani) are 77.5% identical by primary amino acid sequence, are structurally very similar^12–15^ and commonly bind the human angiotensin coverting enzyme 2 (ACE2) protein as a host receptor^1,16^ through their S1_B_ domain. Receptor interaction is known to trigger irreversible conformational changes in coronavirus spike proteins enabling membrane fusion^17^.

## Results

### Identification of SARS-CoV-2 reactive antibodies

In order to identify SARS-CoV-2-neutralizing antibodies, ELISA-(cross)reactivity was assessed of antibody-containing supernatants of a collection of 51 SARS-S hybridoma’s derived from immunized transgenic H2L2 mice that encode chimeric immunoglobulins with human variable heavy and light chains and constant regions of rat origin (Supplementary Table 1). Four of 51 SARS-S hybridoma supernatants displayed ELISA-cross-reactivity with the SARS2-S1 subunit (S residues 1–681; Supplementary Table 1), of which one (47D11) exhibited cross-neutralizing activity of SARS-S and SARS2-S pseudotyped VSV infection. The chimeric 47D11 H2L2 antibody was reformatted to a fully human immunoglobulin, by cloning of the human variable heavy and light chain regions into a human IgG1 isotype backbone. The recombinantly expressed human 47D11 was used for further characterization.

### Antiviral and biochemical properties of the human mAb 47D11

**Fig. 1:**
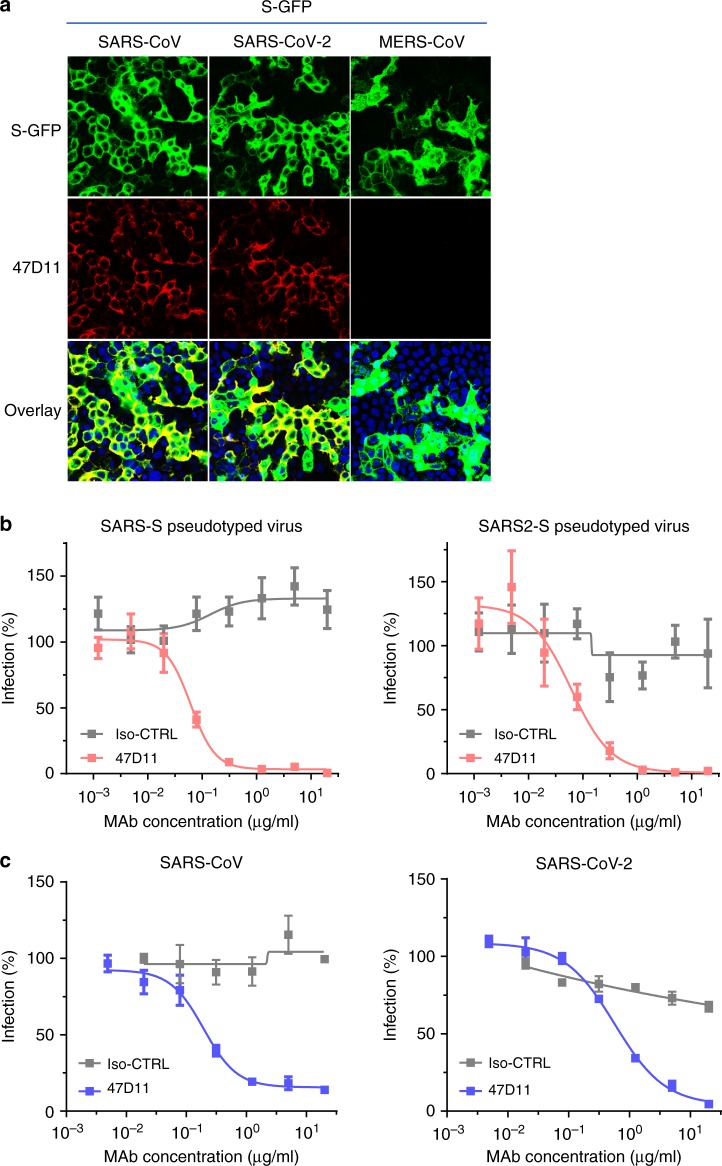
47D11 neutralizes SARS-CoV and SARS-CoV-2. **a** Binding of 47D11 to HEK-293T cells expressing GFP-tagged spike proteins of SARS-CoV and SARS-CoV-2 detected by immunofluorescence assay. The human mAb 7.7G6 targeting the MERS-CoV S1_B_ spike domain was taken along as a negative control, cell nuclei in the overlay images are visualized with DAPI. **b** Antibody-mediated neutralization of infection of luciferase-encoding VSV particles pseudotyped with spike proteins of SARS-CoV and SARS-CoV-2. Pseudotyped VSV particles pre-incubated with antibodies at indicated concentrations (see Methods) were used to infect VeroE6 cells and luciferase activities in cell lysates were determined at 24 h post transduction to calculate infection (%) relative to non-antibody-treated controls. The average ± SD from at least three independent experiments with technical triplicates is shown. Iso-CTRL: an anti-Strep-tag human monoclonal antibody^11^ was used as an antibody isotype control. **c** Antibody-mediated neutralization of SARS-CoV and SARS-CoV-2 infection on VeroE6 cells. The experiment was performed with triplicate samples, the average ± SD is shown. Source data are provided as a Source Data file.

**Fig. 2:**
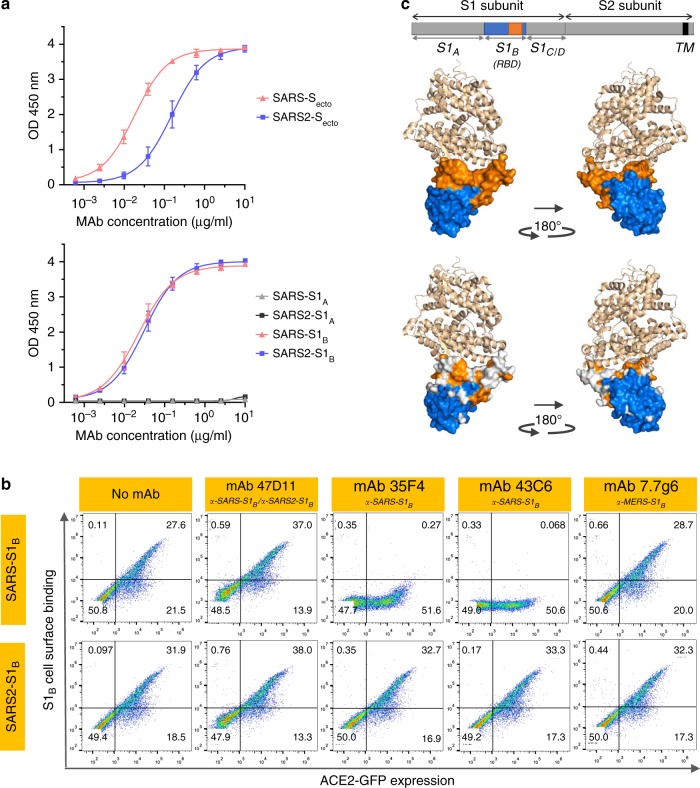
The neutralizing 47D11 mAb binds SARS1-S and SARS2-S RBD without eliminating receptor interaction. **a** ELISA-binding curves of 47D11 to S_ecto_ (upper panel) or S1_A_ and S1_B_ (RBD: receptor-binding domain) (lower panel) of SARS-S and SARS2-S coated at equimolar concentrations. The average ± SD from two independent experiments with technical duplicates is shown. **b** Interference of antibodies with binding of the S-S1_B_ of SARS-CoV and SARS-CoV-2 to cell surface ACE2-GFP analyzed by flow cytometry. Prior to cell binding, S1_B_ was mixed with mAb (mAbs 47D11, 35F4, 43C6, 7.7G6, in H2L2 format) with indicated specificity in a mAb:S1_B_ molar ratio of 8:1 (see Supplementary Fig. 3 for an extensive analysis using different mAb:S1_B_ molar ratio’s). Cells are analyzed for (ACE2-)GFP expression (*x* axis) and S1_B_ binding (*y* axis). Percentages of cells that scored negative, single positive, or double positive are shown in each quadrant. Experiment was done twice, a representative experiment is shown. **c** Divergence in surface residues in S1_B_ of SARS-CoV and SARS-CoV-2. Upper panel: Structure of the SARS-CoV spike protein S1_B_ RBD in complex with human ACE2 receptor (PDB: 2AJF)^24^. ACE2 (wheat color) is visualized in ribbon presentation. The S1_B_ core domain (blue) and subdomain (orange) are displayed in surface presentation using PyMOL, and are visualized with the same colors in the linear diagram of the spike protein above, with positions of the S1 and S2 subunits, the S ectodomain (S_ecto_), the S1 domains S1_A-D_ and the transmembrane domain (TM) indicated. Lower panel: similar as panel above with surface residues on S1_B_ of SARS-CoV that are at variance with SARS-CoV-2 colorored in white. Source data are provided as a Source Data file.

The human 47D11 antibody binds to cells expressing the full-length spike proteins of SARS-CoV and SARS-CoV-2 (Fig. 1a). The 47D11 antibody was found to potently inhibit infection of VeroE6 cells with SARS-S and SARS2-S pseudotyped VSV with IC_50_ values of 0.061 and 0.061 μg/ml (Fig. 1b), respectively. Authentic infection of VeroE6 cells with SARS-CoV and SARS-CoV-2 was neutralized with IC_50_ values of 0.19 and 0.57 μg/ml (Fig. 1c). Using ELISA 47D11 was shown to target the S1_B_ receptor-binding domain (RBD) of SARS-S and SARS2-S. 47D11 bound the S1_B_ of both viruses with similar affinities as shown by the ELISA-based half maximal effective concentration (EC_50_) values (0.02 and 0.03 μg/ml, respectively; Fig. 2a). ELISA-based binding affinity of 47D11 for the spike ectodomain (S_ecto_) of SARS-CoV was higher relative to that of SARS-CoV-2 (EC_50_ values: 0.018 and 0.15 μg/ml, respectively), despite equimolar antigen coating (Supplementary Fig. 1). Congruent with the ELISA-reactivities, measurement of binding kinetics of 47D11 by biolayer interferometry showed that 47D11 binds SARS-S_ecto_ with higher affinity (equilibrium dissociation constant [*K*_*D*_]: 0.745 nM) relative to SARS2-S_ecto_ (*K*_*D*_ 10.8 nM), whereas affinity for SARS-S1_B_ and SARS2-S1_B_ was in a similar range (16.1 and 9.6 nM, respectively, Supplementary Fig. 2). This difference may originate from differences in epitope accessibility in SARS-S versus SARS2-S, as domain B can adopt a closed and open conformation in the prefusion spike homotrimer^12,13^. Remarkably, binding of 47D11 to SARS-S1_B_ and SARS2-S1_B_ did not compete with S1_B_ binding to the ACE2 receptor expressed at the cell surface as shown by flow cytometry (Fig. 2b; Supplementary Fig. 3) nor with S_ecto_ and S1_B_ binding to soluble ACE2 in solid-phase based assay (Supplementary Fig. 4), whereas two SARS-S1 specific antibodies 35F4 and 43C6 that neutralize SARS-S (but not SARS2-S) pseudotyped VSV infection (Supplementary Fig. 5) do block binding of SARS-S_ecto_ and SARS-S1_B_ to ACE2. Using a trypsin-triggered cell-cell fusion assay, 47D11 was shown to impair SARS-S and SARS2-S mediated syncytia formation (Supplementary Fig. 6). Our data show that 47D11 neutralizes SARS-CoV and SARS-CoV-2 through a yet unknown mechanism that is different from receptor-binding interference. Alternative mechanisms of coronavirus neutralization by RBD-targeting antibodies have been reported including spike inactivation through antibody-induced destabilization of its prefusion structure^17^, which may also apply for 47D11.

### 47D11 targets a conserved epitope in the SARS2-S-S1_B_ domain

The SARS2-S1_B_ RBD (residues 338–506) consists of a core domain and a receptor-binding subdomain (residues 438–498) looping out from the antiparallel betasheet core domain structure that directly engages the receptor. Compared to the S1_B_ core domain, the protein sequence identity of the S1_B_ receptor interacting subdomain of SARS-S and SARS2-S is substantially lower (46.7% versus 86.3%; Supplementary Fig. 7 and Fig. 2c). Potent neutralizing antibodies often target this receptor-binding subdomain. However, due to common variations in this subdomain, these antibodies are often virus-specific and bind and neutralize related viruses poorly^18,19^. The cross-reactive nature of 47D11 indicates that the antibody is more likely to target the conserved core structure of the S1_B_ RBD. Interestingly, the SARS-CoV-neutralizing antibody CR3022 also targeting the S1_B_ core domain was recently found to cross-bind SARS-CoV-2, though its ability to cross-neutralize SARS-CoV-2 infection was not reported^18,20^. S1_B_ binding by 47D11 further away from the receptor-binding interface explains its inability to compromise spike–receptor interaction and opens possibilities for combination treatments with non-competing, potent neutralizing antibodies that target the receptor-binding subdomain. Antibody combinations targeting non-overlapping epitopes may act synergistically resulting in lower dosage and may mitigate risk of immune escape^20^.

In conclusion, this is the first report of a (human) monoclonal antibody that neutralizes SARS-CoV-2. 47D11 binds a conserved epitope on the spike RBD explaining its ability to cross-neutralize SARS-CoV and SARS-CoV-2, using a mechanism that is independent of receptor-binding inhibition. This antibody will be useful for development of antigen detection tests and serological assays targeting SARS-CoV-2. Neutralizing antibodies can alter the course of infection in the infected host supporting virus clearance or protect an uninfected host that is exposed to the virus^4^. Hence, this antibody—either alone or in combination—offers the potential to prevent and/or treat COVID-19, and possibly also other future emerging diseases in humans caused by viruses from the *Sarbecovirus* subgenus.

## Methods

### Expression and purification of coronavirus spike proteins

Coronavirus spike ectodomains (S_ecto_) of SARS-CoV-2 (residues 1–1213; strain Wuhan-Hu-1; GenBank: QHD43416.1) and HCoV-OC43 (residues 15–1263; strain Paris; UniProtKB: Q696P8) were expressed transiently in HEK-293T cells with a C-terminal trimerization motif and Strep-tag using the pCAGGS expression plasmid. Similarly, pCAGGS expression vectors encoding S1 or its subdomains of SARS-CoV (S1, residues 1–676; S1_A_, residues 1–302; S1_B_, residues, 325–533), and SARS-CoV-2 (S1, residues 1–682; S1_A_, residues 1–294; S1_B_, residues 329–538) C-terminally tagged with Fc domain of human or mouse IgG or strep-tag were generated as described before^21^. Coronavirus spike ectodomain of MERS-CoV (residues 19–1262; strain EMC; GenBank: YP_009047204.1) and SARS-CoV (residues 15–1182; strain Urbani; GeneBank: AY278741.1) fused with an C-terminal trimerization motif, a thrombin cleavage site and a strep-tag purification tag were in-frame cloned into pMT\Bip\V5\His expression vector. Furin cleavage site at the S1/S2 junction was mutated to prevent cleavage by furin at this position. Spike ectodomains were stably produced in *Drosophila* S2 cell line, as previously described^22^. Recombinant proteins were affinity purified from the culture supernatant by protein-A sepharose beads (GE Healthcare, Catalog# 17-0780-01) or streptactin beads (IBA, Catalog# 2-1201-010) purification. Purity and integrity of all purified recombinant proteins was checked by coomassie stained SDS-PAGE.

### Generation of H2L2 mAbs

H2L2 mice were sequentially immunized in 2 weeks intervals with purified S_ecto_ of different CoVs in the following order: HCoV-OC43, SARS-CoV, MERS-CoV, HCoV-OC43, SARS-CoV, and MERS-CoV. Antigens were injected at 20–25 μg/mouse using Stimune Adjuvant (Prionics) freshly prepared according to the manufacturer's instruction for first injection, whereas boosting was done using Ribi (Sigma) adjuvant. Injections were done subcutaneously into the left and right groin each (50 μl) and 100 μl intraperitoneally. Four days after the last injection, spleen and lymph nodes are harvested, and hybridomas made by standard method using SP 2/0 myeloma cell line (ATCC#CRL-1581) as a fusion partner. Hybridomas were screened in antigen-specific ELISA and those selected for further development, subcloned and produced on a small scale (100 ml of medium). For this purpose, hybridomas are cultured in serum- and protein-free medium for hybridoma culturing (PFHM-II (1×), Gibco) with addition of non-essential amino acids 100× NEAA, Biowhittaker Lonza, Catalog# BE13-114E). H2L2 antibodies were purified from hybridoma culture supernatants using Protein-G affinity chromatography (Merck KGaA, Catalog# 16-266). Purified antibodies were stored at 4˚C until use. The animal studies were done under the animal permit AVD101002016512, approved by the CCD (central committee for animal experiments).

### Production of human monoclonal antibody 47D11

For recombinant human mAb production, the cDNA’s encoding the 47D11 H2L2 mAb variable regions of the heavy and light chains were cloned into expression plasmids containing the human IgG1 heavy chain and Ig kappa light chain constant regions, respectively (InvivoGen). Both plasmids contain the interleukin-2 signal sequence to enable efficient secretion of recombinant antibodies. Recombinant human 47D11 mAb and previously described isotype control (anti-strep-tag mAb) or 7.7G6 mAb were produced in HEK-293T cells following transfection with pairs of the IgG1 heavy and light chain expression plasmids according to protocols from InvivoGen. Human antibodies were purified from cell culture supernatants using Protein-A affinity chromatography. Purified antibodies were stored at 4˚C until use.

### Immunofluorescence microscopy

Antibody binding to cell surface spike proteins of SARS-CoV, SARS-CoV-2, and MERS-CoV was measured by immunofluoresence microscopy. HEK-293T (ATCC#CRL-3216) cells seeded on glass slides were transfected with plasmids encoding SARS-S, SARS2-S, or MERS-S - C-terminally fused to the green fluorescence protein (GFP) using Lipofectamine 2000 (Invitrogen, Catalog# 11668019). Two days post transfection, cells were fixed by incubation with 2% paraformaldehyde in phosphate-buffered saline (PBS) for 20 min at room temperature and stained for nuclei with 4,6-diamidino-2-phenylindole (Sigma, Catalog# D9542). Cells were subsequently incubated with mAbs at a concentration of 10 µg/ml for 1 hour at room temperature, followed by incubation with 1:200 diluted Alexa Fluor 594 conjugated goat anti-human IgG antibodies (Invitrogen, Thermo Fisher Scientific, Catalog# A-11014) for 45 min at room temperature. The fluorescence images were recorded using a Leica SpeII confocal microscope.

### Flow cytometry-based receptor-binding inhibition assay

Antibody interference of S1_B_ binding to human ACE2 receptor on the cell surface was measured by flow cytometry. HEK-293T cells were seeded at a density of 2.5 × 10^5^ cells per ml in a T75 flask. After reaching 70~80% confluency, cells were transfected with an expression plasmid encoding human ACE2 - C-terminally fused to the GFP using Lipofectamine 2000 (Invitrogen). Two days post transfection, cells were dissociated by cell dissociation solution (Sigma-aldrich, Merck KGaA; Catalog# C5914). In all, 2.5 µg/ml of human Fc tagged SARS-S1_B_ and SARS2-S1_B_ was pre-incubated with mAb at the indicated mAb:S1_B_ molar ratios for 1 hour on ice and subjected to flow cytometry. Single-cell suspensions in FACS buffer were centrifuged at 400 × *g* for 10 min. Cells were subsequently incubated with S1_B_ and mAb mixture for 1 hour on ice, followed by incubation with 1:200 diluted Alexa Fluor 594 conjugated goat anti-human IgG antibodies (Invitrogen, Thermo Fisher Scientific, Catalog# A-11014) for 45 min at room temperature. Cells were subjected to flow cytometric analysis with a CytoFLEX Flow Cytometer (Beckman Coulter). The results were analyzed by FlowJo (version 10). FSC/SSC gates were used to select mononuclear cells. Control antibody staining was used to define positive/negative cell populations.

### Pseudotyped virus neutralization assay

Production of VSV pseudotyped with SARS-S and SARS2-S was performed as described previously with some adaptations^11^. Briefly, HEK-293T cells were transfected with pCAGGS expression vectors encoding SARS-S or SARS2-S carrying a 28- or 18-a.a. cytoplasmic tail truncation, respectively. One day post transfection, cells were infected with the VSV-G pseudotyped VSVΔG bearing the firefly (*Photinus pyralis*) luciferase reporter gene. Twenty-four hours later, supernatants containing SARS-S/SARS2-S pseudotyped VSV particles were harvested and titrated on African green monkey kidney VeroE6 (ATCC#CRL-1586) cells. In the virus neutralization assay, mAbs were fourfold serially diluted at two times the desired final concentration in DMEM supplemented with 1% fetal calf serum (Bodinco), 100 U/ml Penicillin and 100 µg/ml Streptomycin (Lonza, Catalog# 17-602E). Diluted mAbs were incubated with an equal volume of pseudotyped VSV particles for 1 hour at room temperature, inoculated on confluent VeroE6 monolayers in 96-well plate, and further incubated at 37 °C for 24 hours. Luciferase activity was measured on a Berthold Centro LB 960 plate luminometer using D-luciferin as a substrate (Promega). The percentage of infectivity was calculated as ratio of luciferase readout in the presence of mAbs normalized to luciferase readout in the absence of mAb. The half maximal inhibitory concentrations (IC_50_) were determined using 4-parameter logistic regression (GraphPad Prism version 8).

### Virus neutralization assay

Neutralization of authentic SARS-CoV and SARS-CoV-2 was performed using a plaque reduction neutralization test as described earlier, with some modifications^23^. In brief, mAbs were twofold serially diluted in culture medium starting at 40 µg/ml and 50 μl was mixed with 50 μl (500 TCID_50_) SARS-CoV or SARS-CoV-2 for 1 hour. The mixture was then added to VeroE6 cells and incubated for 1 hour, after which the cells were washed and further incubated in medium for 8 hours. The cells were then fixed and stained using a rabbit anti-SARS-CoV serum (Sino Biological) and a secondary peroxidase-labeled goat anti-rabbit IgG (Dako). The signal was developed using a precipitate forming TMB substrate (True Blue, KPL) and the number of infected cells per well were counted using the ImmunoSpot Image analyzer (CTL Europe GmbH). The half maximal inhibitory concentrations (IC_50_) were determined using 4-parameter logistic regression (GraphPad Prism version 8).

### ELISA analysis of antibody binding to CoV spike antigens

NUNC Maxisorp plates (Thermo Scientific) were coated with equimolar antigen amounts at 4 °C overnight. Plates were washed three times with PBS containing 0.05% Tween-20 and blocked with 3% bovine serum albumin (Bio-Connect) in PBS containing 0.1% Tween-20 at room temperature for 2 hours. Fourfolds serial dilutions of mAbs starting at 10 µg/ml (diluted in blocking buffer) were added and plates were incubated for 1 hour at room temperature. Plates were washed three times and incubated with horseradish peroxidase (HRP)-conjugated goat anti-human secondary antibody (ITK Southern Biotech) diluted 1:2000 in blocking buffer for 1 hour at room temperature. An HRP-conjugated anti-StrepMAb (IBA, Catalog# 2-1509-001) antibody was used to corroborate equimolar coating of the strep-tagged spike antigens. HRP activity was measured at 450 nanometer using tetramethylbenzidine substrate (BioFX) and an ELISA plate reader (EL-808, Biotek). Half-maximum effective concentration (EC_50_) binding values were calculated by non-linear regression analysis on the binding curves using GraphPad Prism (version 8).

### Reporting summary

Further information on research design is available in the Nature Research Reporting Summary linked to this article.

## Supplementary information

Supplementary Information

Reporting Summary

## Data Availability

Data underlying Figs. 1b, c, 2a, Supplementary Figs. 1, 2, 4, and 5 are provided as Source Data files. Antibody and antibody sequences are available (by contacting Vincent Rijsman from the Utrecht University Research Support Office; V.M.C.Rijsman@uu.nl) for research purposes only under an MTA, which allows the use of the antibody sequences for non-commercial purposes but not their disclosure to third parties. All other data are available from the corresponding author upon reasonable requests.

## Source data

Source Data
