# Publisher Correction: A human monoclonal antibody blocking SARS-CoV-2 infection

**DOI:** 10.1038/s41467-020-16452-w

**Published:** 2020-05-14

**Authors:** Chunyan Wang, Wentao Li, Dubravka Drabek, Nisreen M. A. Okba, Rien van Haperen, Albert D. M. E. Osterhaus, Frank J. M. van Kuppeveld, Bart L. Haagmans, Frank Grosveld, Berend-Jan Bosch

**Affiliations:** 1 Virology Section, Infectious Diseases and Immunology Division, Department of Biomolecular Health Sciences, Faculty of Veterinary Medicine, Utrecht University, Utrecht, the Netherlands; 2 Department of Cell Biology, Erasmus Medical Center, Rotterdam, the Netherlands; 3 Harbour BioMed, Rotterdam, the Netherlands; 4 Department of Viroscience, Erasmus Medical Center, Rotterdam, the Netherlands; 5 University of Veterinary Medicine, Hannover, Germany

**Keywords:** Immunotherapy, Virology

Correction to: *Nature Communications* 10.1038/s41467-020-16256-y, published online 4 May 2020.

The competing interests section of the original article contained an error. In the sentence “A patent application has been filed on 12 March 2020 on monoclonal antibodies targeting SARS-CoV-2 (United Kingdom patent application no. 2003632.3”, the number 2003632 was hyperlinked in error to an irrelevant page. The link has been removed both from the PDF and the HTML version of the article.

