# Supplementary material for: A human monoclonal antibody blocking SARS-CoV-2 infection
Source: Nat Commun. 2020 May 4;11:2251. doi: 10.1038/s41467-020-16256-y (PMC7198537; doi:10.1038/s41467-020-16256-y)
Supplement: Supplementary file 1 — Supplementary Information [file 41467_2020_16256_MOESM1_ESM.pdf]

Supplemental information

A human monoclonal antibody blocking SARS-CoV-2 infection

Wang et al.

Hybridoma	SARS-S_{ecto}	SARS-S1	SARS-$S1_A$	SARS2-S1
44B3	2,5	2,7	3,3	0,1
45E10	3,0	0,8	1,7	0,0
46F11	2,4	2,7	3,3	0,0
39F9	2,9	3,3	3,5	0,0
41A7	2,6	1,0	1,9	0,0
28 E3	2,4	2,3	3,2	0,0
34C10	1,3	1,0	1,9	0,0
16C10	2,4	0,6	1,7	0,1
14B1	2,6	2,9	3,3	0,1
30B1	0,6	0,5	1,1	0,0
28G10	1,0	1,3	2,6	0,0
28F6	2,4	2,9	3,0	0,0
40H10	1,2	0,7	1,9	0,0
39A4	1,7	1,5	2,8	0,0
37G1	1,3	0,9	1,7	0,0
44E11	2,8	3,3	3,5	0,1
19C1	1,9	0,4	1,2	0,1
58D2	2,6	2,8	3,4	0,1
14C1	2,8	1,2	2,6	0,0
45H1	2,3	3,1	3,6	0,0
24F5	3,3	3,4	3,6	0,0
52D9	1,5	1,6	2,3	1,3
45E6	2,4	2,6	3,3	0,0
47D11	3,4	3,0	0,0	1,5
47G10	2,6	2,8	0,1	0,0
48G1	3,3	3,4	0,1	0,0
49F1	1,8	2,0	0,0	1,3
43C6	3,1	3,4	0,1	0,1
22E10	3,2	3,4	0,1	0,0
28D11	2,7	3,1	0,1	0,0
28H3	2,8	1,8	0,0	0,0
25E7	3,1	3,3	0,1	0,1
22E8	1,2	1,2	0,1	0,0
35F4	3,2	3,6	0,1	0,0
43G5	3,2	3,3	0,1	0,1
47F8	1,4	1,4	0,0	0,0
43B4	3,2	3,3	0,1	0,0
49B10	1,1	0,6	0,0	0,2
51C11	1,9	1,9	0,0	0,0
36F6	1,7	2,7	0,1	0,3
65H8	3,2	3,3	0,1	0,1
65H9	1,6	1,7	0,1	2,5
48D5	3,3	3,5	0,1	0,0
35E2	2,5	3,3	0,2	0,0
44G3	2,4	2,8	0,1	0,0
9H9	1,8	0,1	0,0	0,1
25C3	3,0	0,1	0,1	0,1
29E6	1,1	0,1	0,1	0,0
43F11	2,8	0,1	0,1	0,0
47C4	1,5	0,0	0,1	0,0
13F11	3,0	0,0	0,0	0,0

ELISA reactivity hybr. sups	# hybr sups
anti-SARS-$S1_A$	23
anti-SARS-S1 (but not binding $S1_A$)	22
anti-SARS-S_{ecto} (but not binding S1)	6
**Total**	**51**

Supplementary Table 1. ELISA cross-reactivity of antibody-containing supernatants of SARS-S H2L2 hybridomas towards SARS2-S1. SARS-S targeting hybridomas were developed by conventional hybridoma technology from immunized H2L2 transgenic mice (Harbour Biomed)[1]. These mice - carrying genes encoding the heavy and light chain human immunoglobulin repertoire - were sequentially immunized with 2-week intervals with trimeric spike protein ectodomains (S_{ecto}) of three human coronaviruses from the betacoronavirus genus in the following order: 1. HCoV-OC43-S_{ecto}, 2. SARS-CoV-S_{ecto}, 3. MERS-CoV-S_{ecto}, 4. HCoV-OC43-S_{ecto}, 5. SARS-CoV-S_{ecto}, 6. MERS-CoV-S_{ecto}. Four days after the last immunization, splenocytes and lymph node lymphocytes were harvested and hybridomas were generated. Antibodies in the cell supernatants were tested for ELISA-reactivity against SARS-S_{ecto}, SARS-S1, SARS-$S1_A$ and SARS2-S1. Of the 51 hybridoma supernatants that reacted with SARS-S_{ecto} only, 23 reacted with SARS-$S1_A$, 22 with SARS-S1 but not SARS-$S1_A$, 6 with SARS-S_{ecto} but not SARS-S1. Four of the 51 SARS-S_{ecto} hybridoma supernatants reacted with SARS2-S1 (see column on the right). The table displays ELISA-signal intensities (OD_{450nm} values) of hybridoma supernatants for the different antigens.

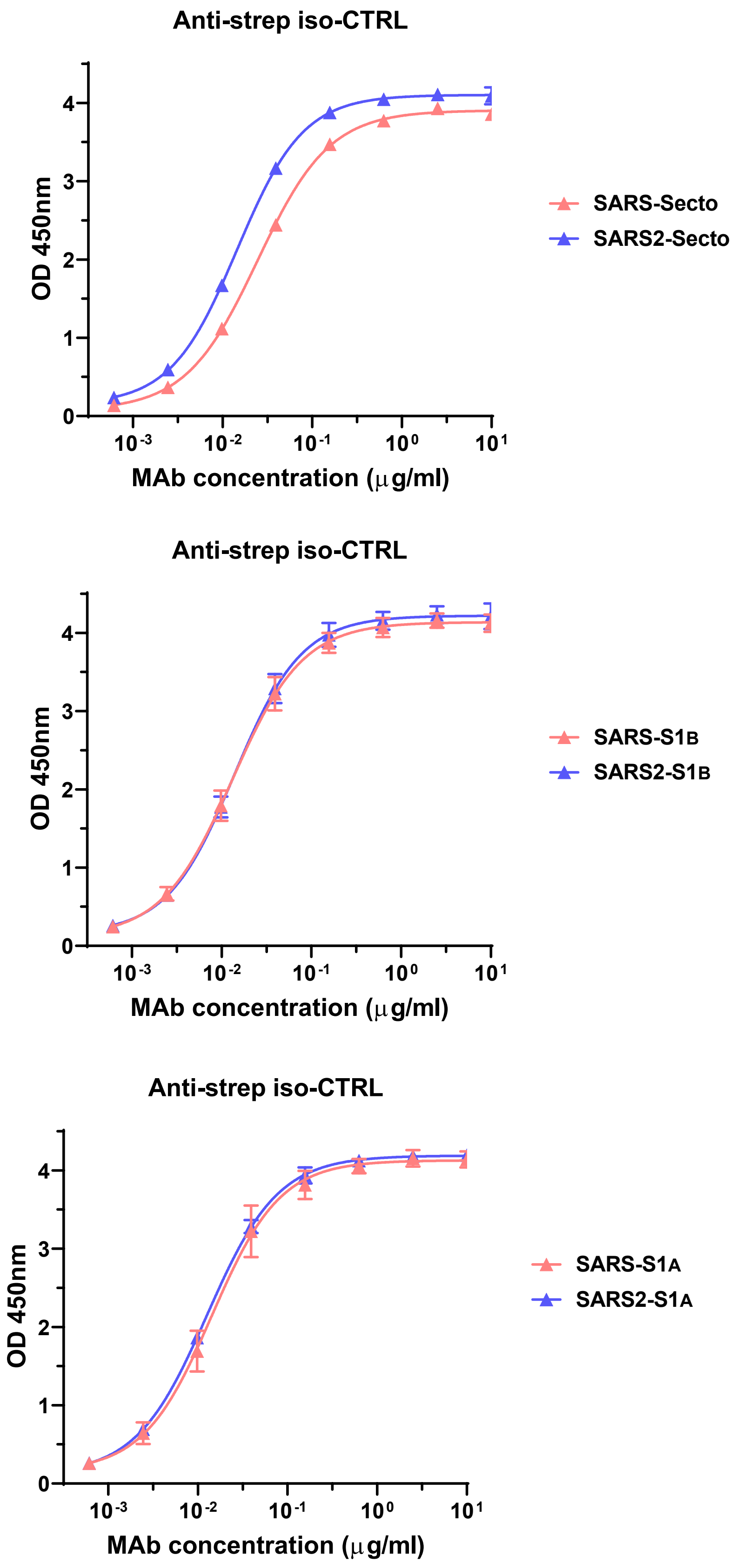

Supplementary Figure 1. ELISA binding curve of the anti-StrepMAb (IBA) antibody to Strep-tagged spike antigens to corroborate equimolar ELISA plate coating of SARS-S_{ecto} / SARS2-S_{ecto} (upper panel), SARS-$S1_B$ / SARS2-$S1_B$ (middle panel) and SARS-$S1_A$ / SARS2-$S1_A$ (lower panel) antigens used in Fig.2a. The average ± SD from two independent experiments with technical duplicates is shown. Source data are provided as a Source Data file.

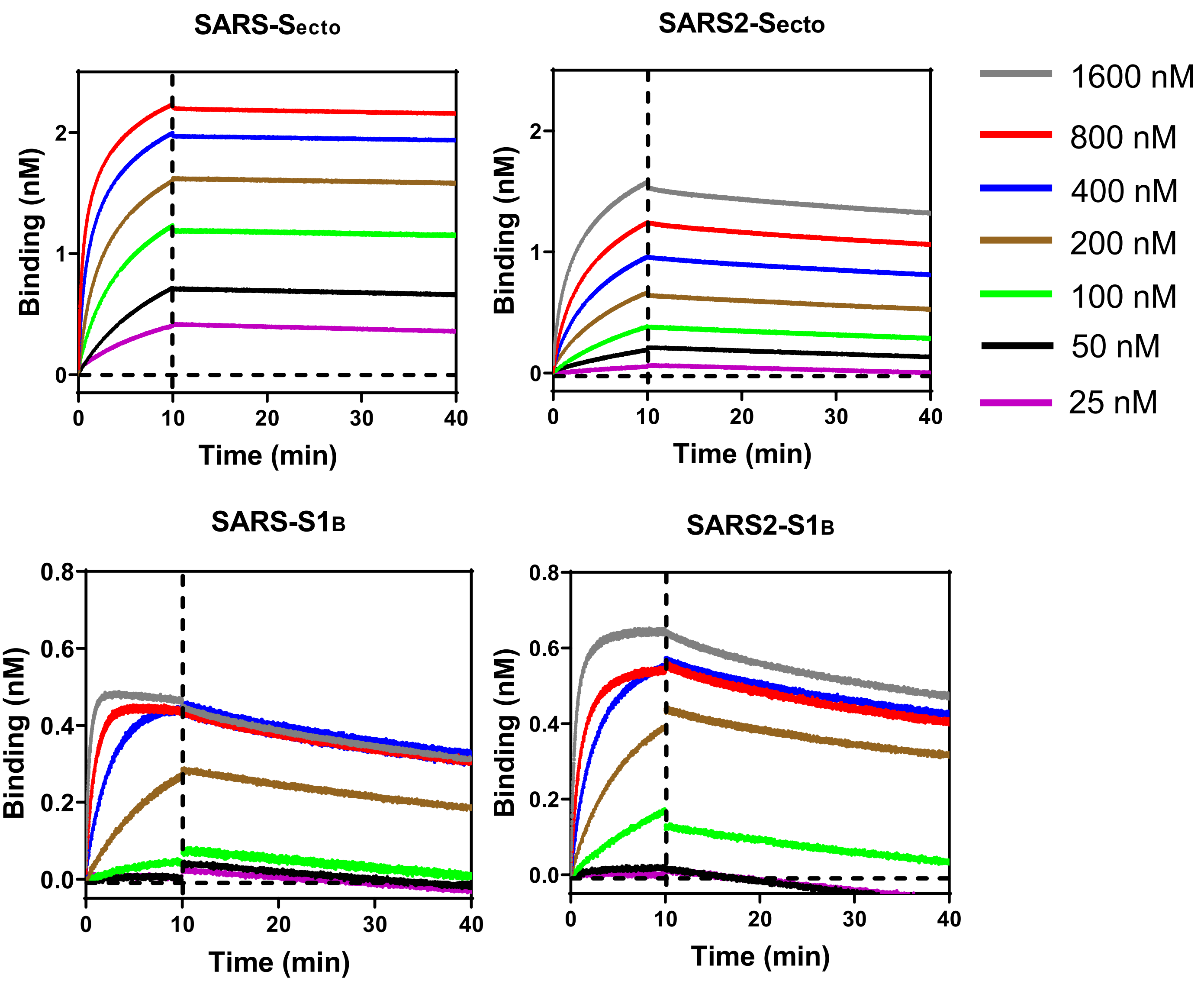

Spike protein	K_D (nM)	K_{on} ($M^{-1}sec^{-1}$)	K_{off} (sec^{-1})
SARS-Secto	0.745 (± 0.532)	3.75 (± 1.27) × 10^4	3.42 (± 3.34) × 10^{-5}
SARS2-Secto	10.8 (± 2.46)	9.77 (± 4.09) × 10^3	9.85 (± 3.61) × 10^{-5}
SARS-S1$_B$	16.1 (± 13.3)	2.00 (± 0.961) × 10^4	2.10 (± 0.239) × 10^{-4}
SARS2-S1$_B$	9.56 (± 2.68)	1.51 (± 0.285) × 10^4	1.37 (± 0.0728) × 10^{-4}

Supplementary Figure 2. Binding kinetics of 47D11 to the S ectodomain and S1$_B$ of SARS-CoV and SARS-CoV-2. Binding kinetics of 47D11 to immobilized recombinant SARS-S$_{ecto}$, SARS2-S$_{ecto}$, SARS-S1$_B$ and SARS2-S1$_B$ was measured using biolayer interferometry at 25°C[2]. Kinetic binding assay was performed by loading 47D11 mAb at optimal concentration (42 nM) on anti-human Fc biosensor for 10 mins. Antigen association step was performed by incubating the sensor with a range of concentrations of the recombinant spike ectodomain (1600-800-400-200-100-50-25 nM) for 10 min, followed by a dissociation step in PBS for 60 min. The kinetics constants were calculated using 1:1 Langmuir binding model on Fortebio Data Analysis 7.0 software. The experiment was performed twice, data from a representative experiment are shown. Source data are provided as a Source Data file.

A

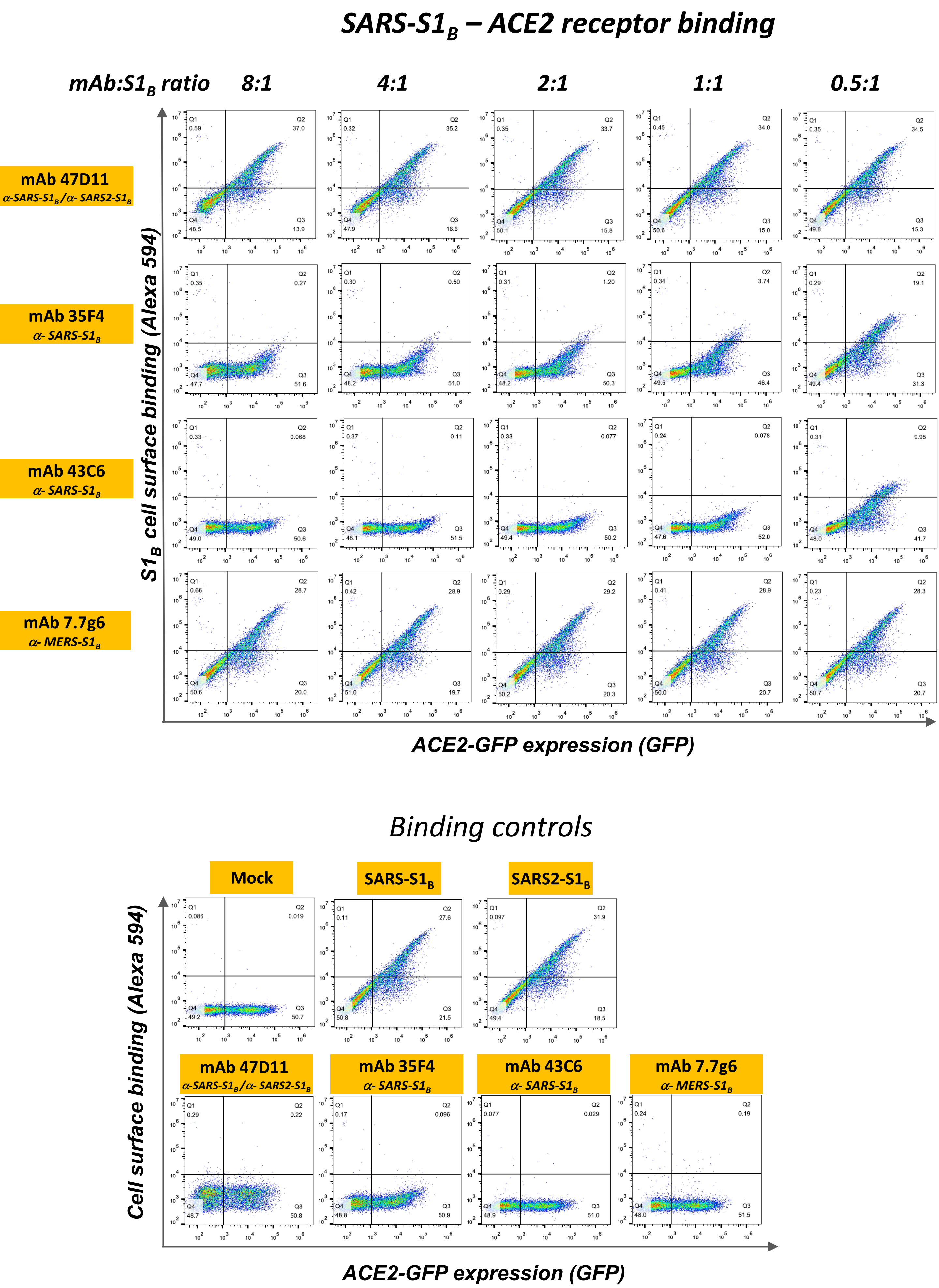

SARS-S1B – ACE2 receptor binding
mAb:S1B ratio
8:1
4:1
2:1
1:1
0.5:1
mAb 47D11
α-SARS-S1B/α- SARS2-S1B
Q1 0.59 Q2 37.0 Q4 48.5 Q3 13.9
Q1 0.32 Q2 35.2 Q4 47.9 Q3 16.6
Q1 0.35 Q2 33.7 Q4 50.1 Q3 15.8
Q1 0.45 Q2 34.0 Q4 50.6 Q3 15.0
Q1 0.35 Q2 34.5 Q4 49.8 Q3 15.3
mAb 35F4
α- SARS-S1B
Q1 0.35 Q2 0.27 Q4 47.7 Q3 51.6
Q1 0.30 Q2 0.50 Q4 48.2 Q3 51.0
Q1 0.31 Q2 1.20 Q4 48.2 Q3 50.3
Q1 0.34 Q2 3.74 Q4 49.5 Q3 46.4
Q1 0.29 Q2 19.1 Q4 49.4 Q3 31.3
mAb 43C6
α- SARS-S1B
Q1 0.33 Q2 0.068 Q4 49.0 Q3 50.6
Q1 0.37 Q2 0.11 Q4 48.1 Q3 51.5
Q1 0.33 Q2 0.077 Q4 49.4 Q3 50.2
Q1 0.24 Q2 0.078 Q4 47.6 Q3 52.0
Q1 0.31 Q2 9.95 Q4 48.0 Q3 41.7
mAb 7.7g6
α- MERS-S1B
Q1 0.66 Q2 28.7 Q4 50.6 Q3 20.0
Q1 0.42 Q2 28.9 Q4 51.0 Q3 19.7
Q1 0.29 Q2 29.2 Q4 50.2 Q3 20.3
Q1 0.41 Q2 28.9 Q4 50.0 Q3 20.7
Q1 0.23 Q2 28.3 Q4 50.7 Q3 20.7
S1B cell surface binding (Alexa 594)
ACE2-GFP expression (GFP)
Binding controls
Mock
Q1 0.086 Q2 0.019 Q4 49.2 Q3 50.7
SARS-S1B
Q1 0.11 Q2 27.6 Q4 50.8 Q3 21.5
SARS2-S1B
Q1 0.097 Q2 31.9 Q4 49.4 Q3 18.5
mAb 47D11
α-SARS-S1B/α- SARS2-S1B
Q1 0.29 Q2 0.22 Q4 48.7 Q3 50.8
mAb 35F4
α- SARS-S1B
Q1 0.17 Q2 0.096 Q4 48.8 Q3 50.9
mAb 43C6
α- SARS-S1B
Q1 0.077 Q2 0.029 Q4 48.9 Q3 51.0
mAb 7.7g6
α- MERS-S1B
Q1 0.24 Q2 0.19 Q4 48.0 Q3 51.5
Cell surface binding (Alexa 594)
ACE2-GFP expression (GFP)

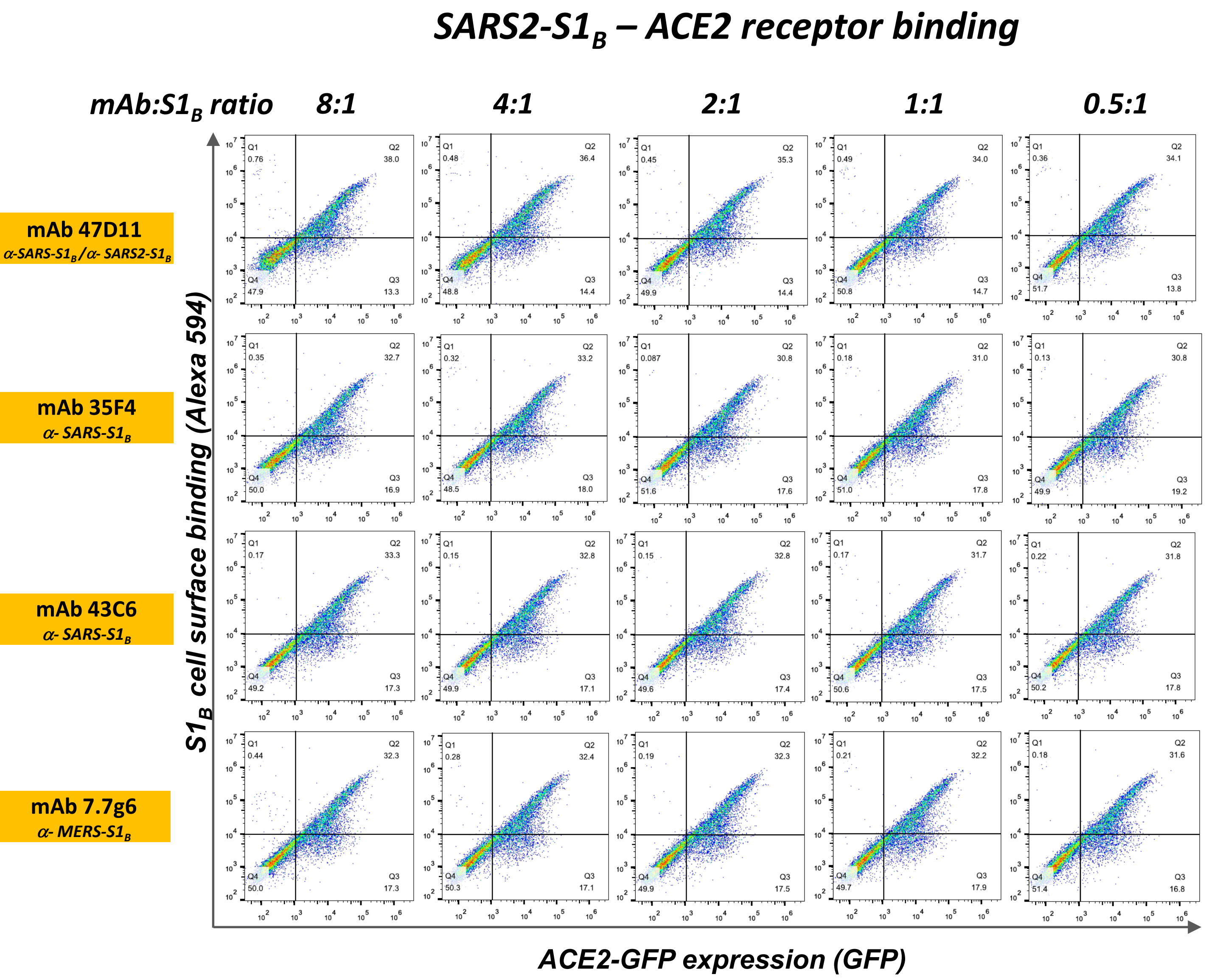

SARS2-S1B – ACE2 receptor binding
mAb:S1B ratio
8:1
4:1
2:1
1:1
0.5:1
mAb 47D11
α-SARS-S1B/α- SARS2-S1B
mAb 35F4
α- SARS-S1B
mAb 43C6
α- SARS-S1B
mAb 7.7g6
α- MERS-S1B
S1B cell surface binding (Alexa 594)
ACE2-GFP expression (GFP)

B

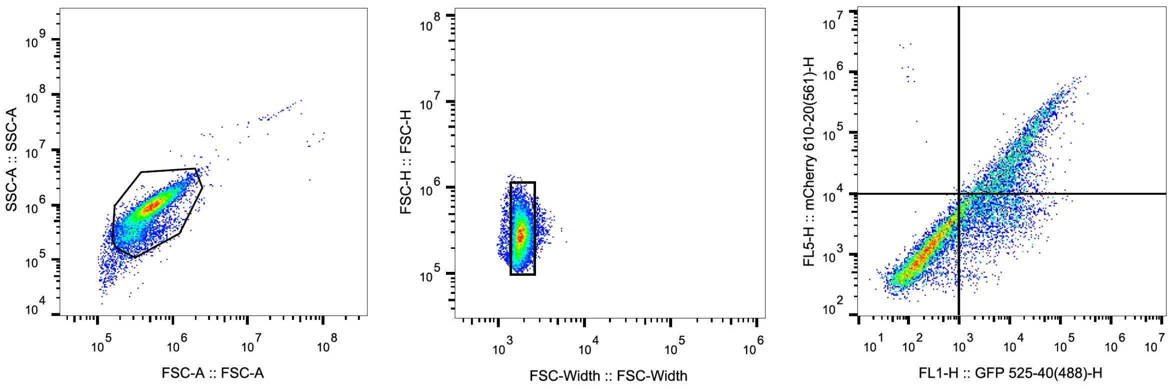

Supplementary Figure 3. 47D11 does not prevent binding of SARS-S1$_B$ and SARS2-S1$_B$ to ACE2-expressing cells. A) Human HEK-293T cells expressing human ACE2-GFP proteins (see Methods) were detached and fixed with 2% PFA, incubated with a fixed amount of human Fc-tagged S1$_B$ domain of SARS-S or SARS2-S that was preincubated for 1h with mAb (mAbs 47D11, 35F4, 43C6, 7.7G6, in H2L2 format) at the indicated mAb:S1$_B$ molar ratios, and analysed by flow cytometry using a Alexa Fluor 594-conjugated secondary antibody targeting the human Fc tag. Cells are analysed for GFP expression (x-axis, GFP signal) and antibody binding (y-axis, Alexa 594 signal). Percentages of cells that scored negative, single positive, or double positive are shown in each quadrant. Binding controls include PBS-treated cells (mock), treatment of cells with SARS-S1$_B$ and SARS2-S1$_B$ in the absence of antibody, and cells treated with antibodies only. The experiment was performed twice, data from a representative experiment are shown. B) Graphical account for FACS sequential gating strategies. FACS gating was performed as previously described[2]. Cells were selected in FSC/SSC dot plot to remove debris, single cells were gated using the FSC-H/FSC-W dot plot. GFP+, Alexa 594+ cells were gated and compared with a control sample with no detectable fluorochrome expression.

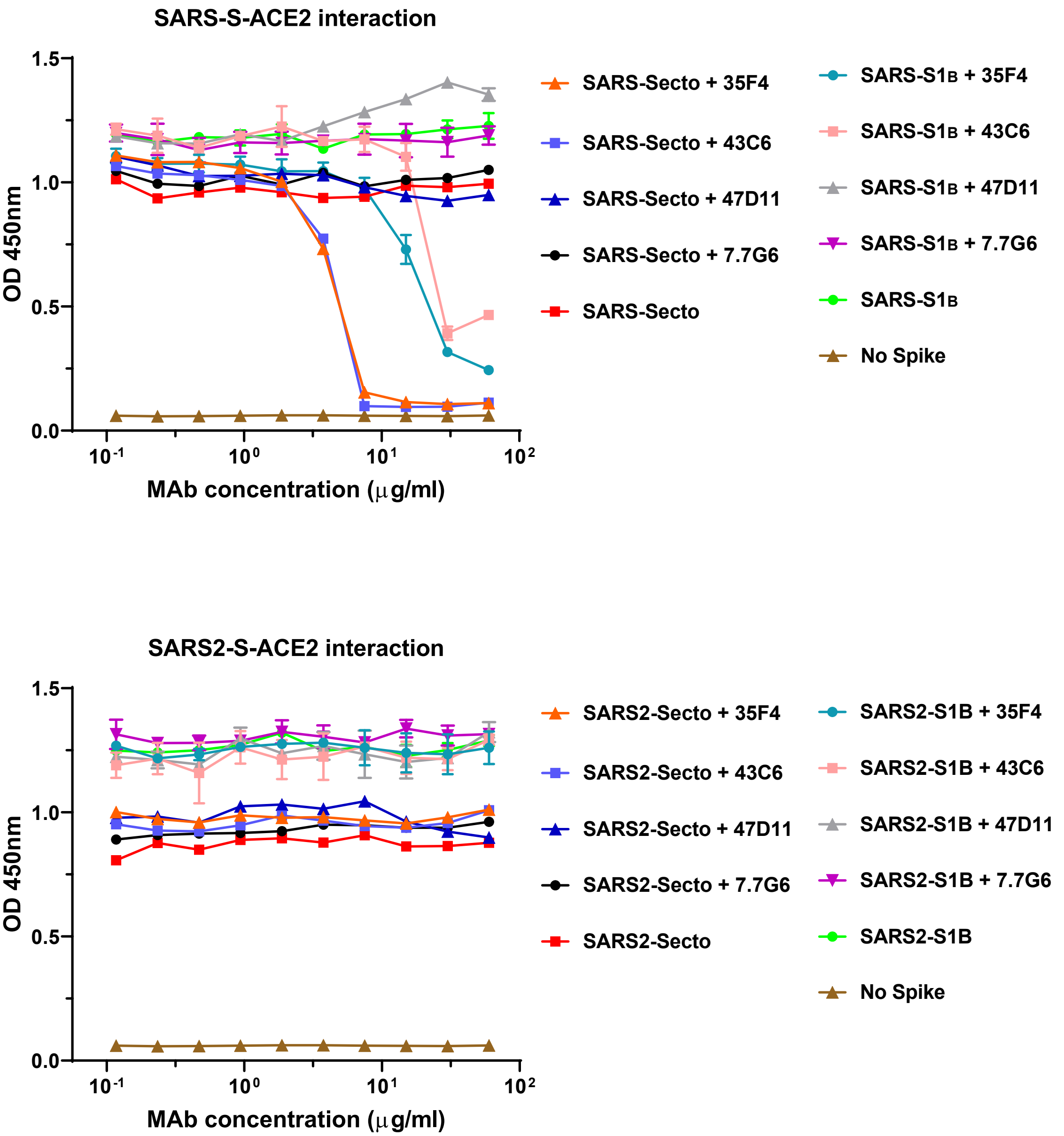

Supplementary Figure 4. ELISA-based receptor binding inhibition assay. The ELISA-based receptor binding inhibition assay was performed as described previously with some adaptations[1]. Recombinant soluble human ACE2 was coated on NUNC Maxisorp plates (Thermo Scientific) at 4°C overnight. Plates were washed three times with PBS containing 0.05% Tween-20 and blocked with 5% protifar in PBS containing 0.1% Tween-20 at room temperature for 3 hours. Recombinant S_{ecto} and $S1_B$ of SARS-S or SARS2-S (300 ng) and serially diluted mAbs (mAbs 47D11, 35F4, 43C6, 7.7G6, in H2L2 format) were mixed for 1h at RT, added to the plate for 2 hour on ice, after which the plates were washed three times. Binding to ACE2 was detected using HRP-conjugated StrepMAb (IBA) that recognizes the C-terminal Streptag on the S_{ecto} and $S1_B$ proteins. The experiment was performed twice. The average ± SD from a representative experiment with technical duplicates is shown. Source data are provided as a Source Data file.

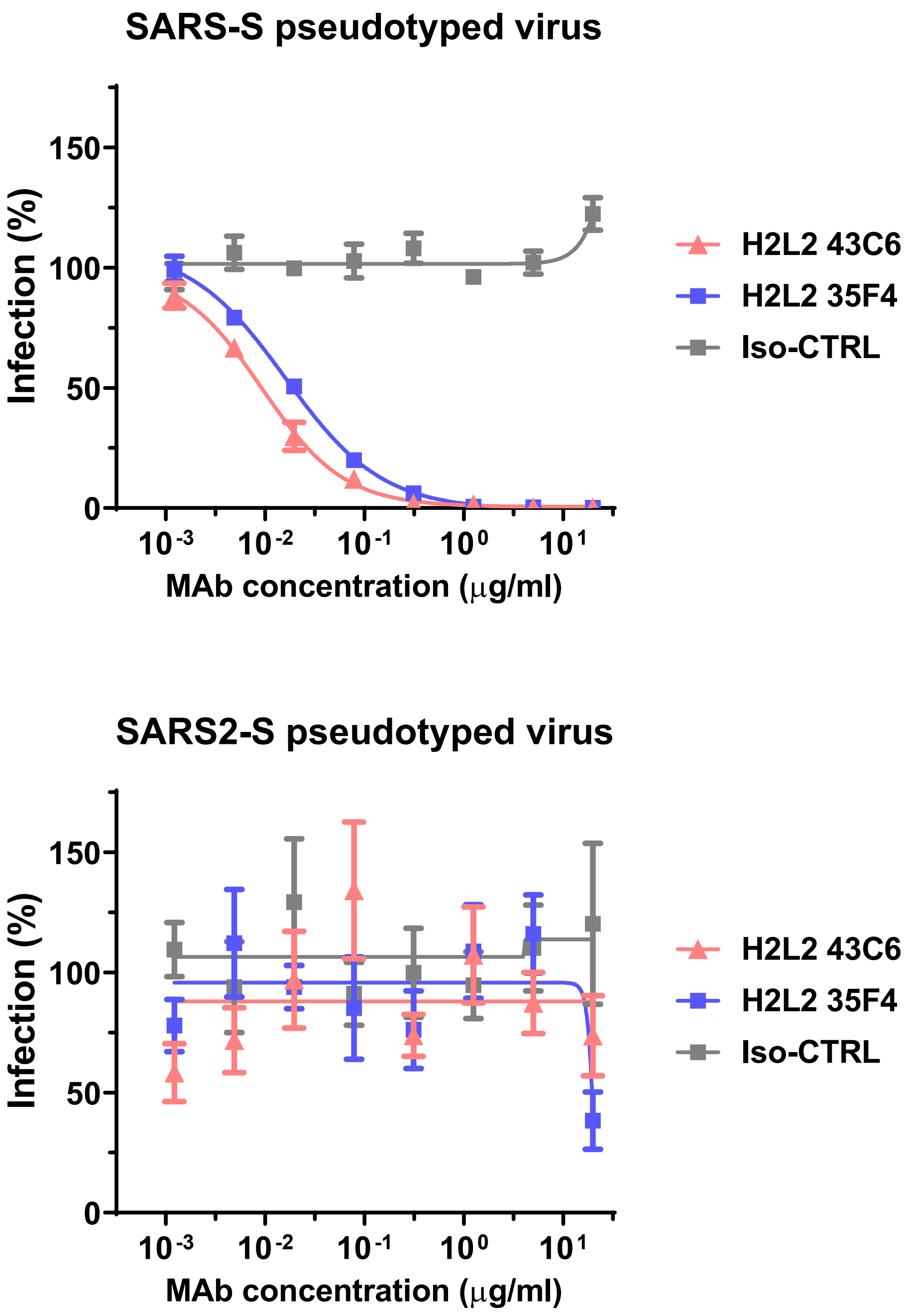

Supplementary Figure 5. H2L2 monoclonal antibodies 35F4 and 43C6 neutralize SARS-CoV but not SARS-CoV-2. Antibody-mediated neutralization of infection of VSV particles pseudotyped with spike proteins of SARS-CoV (upper panel) and SARS-CoV-2 (lower panel) by the 35F4 and 43C6 H2L2 antibodies targeting SARS-S1 but not SARS2-S1 (see Suppl.Fig.1). An irrelevant antibody was taken along as a human IgG1 isotype control. The average ± SD from two independent experiments with technical triplicates is shown. Source data are provided as a Source Data file.

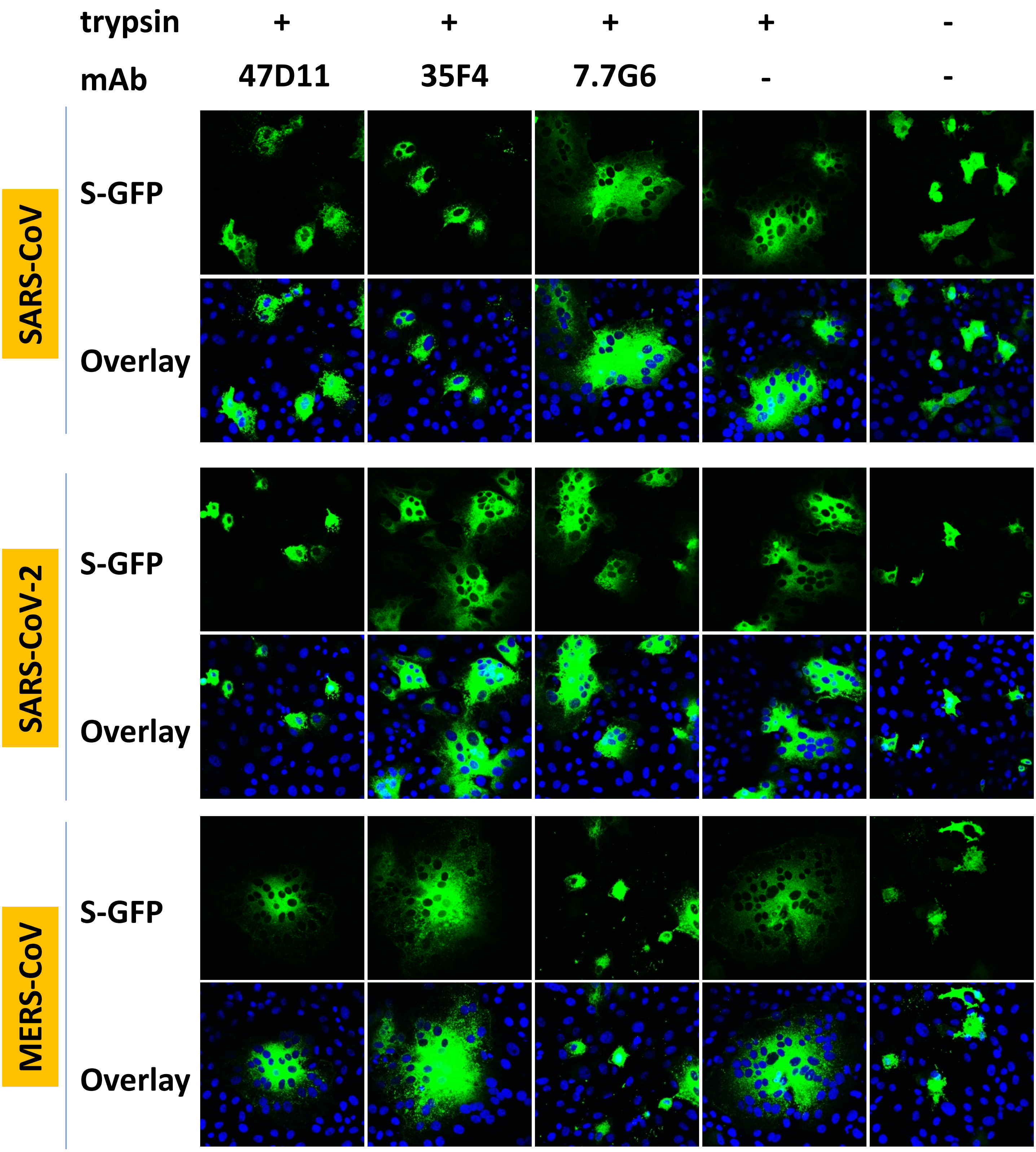
trypsin
+
+
+
+
-
mAb
47D11
35F4
7.7G6
-
-
SARS-CoV
S-GFP
Overlay
SARS-CoV-2
S-GFP
Overlay
MERS-CoV
S-GFP
Overlay

Supplementary Figure 6. Cell-cell fusion inhibition assay. The cell-cell-fusion inhibition assay was performed as described previously with some adaptations[1]. VeroE6 cells were seeded with density of 10^5 cells per ml. After reaching 70~80% confluency, cells were transfected with plasmids encoding full length SARS-S, SARS2-S and MERS-S – C-terminally fused to GFP - using Lipofectamine 2000 (Invitrogen). The furin recognition sites in the SARS2-S (R^{682}RAR to A^{682}AAR) and MERS-S (R^{747}SVR to Y^{747}SVR) were mutated to inhibit cleavage of the protein by endogenous furin and allow trypsin-induced syncytia formation. Two days post transfection, cells were pretreated DMEM only or DMEM with 20 μg/ml mAbs for 1 h and subsequently treated with DMEM with 15 μg/ml trypsin (to activate the spike fusion function) in the absence or presence of 20 μg/ml mAbs (47D11 cross-reactive to SARS-S and SARS2-S, 35F4 reactive to SARS-S, 7.7G6 reactive to MERS-S). After incubation at 37°C for 2 hrs, the cells were fixed with 2% PFA in PBS for 20 min at room temperature and stained for nuclei with 4,6-diamidino-2-phenylindole (DAPI). Cells expressing the S-GFP proteins were detected by fluorescence microscopy and S-mediated cell-cell fusion was observed by the formation of (fluorescent) multi-nucleated syncytia. The fluorescence images were recorded using a Leica SpeII confocal microscope. The experiment was performed twice, data from a representative experiment are shown.

```
SARS-RBD    323 CPFGEVFNATKFPSVYAWERKKISNCVADYSVLYNSTFFSTFKCYGVSATKLNDLCFSNV
SARS2-RBD   438 CPFGEVFNATRFASVYAWNRKRISNCVADYSVLYNSASFSTFKCYGVSPTKLNDLCFTNV
                **********:* *****:**:**************: ********** ********:**

SARS-RBD    383 YADSFVVKGDDVRQIAPGQTGVIADYNYKLPDDFMGCVLAWNTRNIDATSTGNYNYKYRY
SARS2-RBD   396 YADSFVIRGDEVRQIAPGQTGKIADYNYKLPDDFTGCVIAWNSNNLDSKVGGNYNYLYRL
                ******::**:********** ************ ***:***:.*:*:.  ***** ** 

SARS-RBD    443 LRHGKLRPFERDISNVPFSPDGKPCTP-PALNCYWPLNDYGFYTTTGIGYQPYRVVVLSF
SARS2-RBD   456 FRKSNLKPFERDISTEIYQAGSTPCNGVEGFNCYFPLQSYGFQPTNGVGYQPYRVVVLSF
                :*:.:*:*******.  :. ...**.   .:***:**:.***  *.*:************

SARS-RBD    502 ELLNAPATVCGP
SARS2-RBD   516 ELLHAPATVCGP
                ***:********
```

Supplementary Figure 7. Protein sequence alignment of the $S1_B$ receptor binding domain (RBD) of the SARS-CoV and SARS-CoV-2 spike proteins by ClustalW. Numbering denotes the residue position in the full-length spike protein of SARS-CoV (Genbank: AAP13441.1) and SARS-CoV-2 (Genbank: QHD43416.1). Asterisks (*) indicated fully conserved residues, the colon symbol (:) indicates conservation between groups of very similar properties, and the period symbol (.) indicates conservation between groups of weakly similar properties. Sequences corresponding to the $S1_B$ receptor binding core domain and the receptor binding subdomain are colored in blue and orange, respectively. The fourteen residues that are involved in binding of SARS-CoV $S1_B$ to human ACE2 are highlighted in grey[3].

Supplementary References

1. Widjaja, I. et al. Towards a solution to MERS: protective human monoclonal antibodies targeting different domains and functions of the MERS-coronavirus spike glycoprotein. Emerg. Microbes Infect. 8, 516-530 (2019).
2. Lenos, K. J. et al. Stem cell functionality is microenvironmentally defined during tumour expansion and therapy response in colon cancer. Nat. Cell Biol. 20, 1193-1202 (2018).
3. Li, F. et al. Structure of SARS coronavirus spike receptor-binding domain complexed with receptor. Science 309, 1864-1868 (2005).
