# Supplementary material for: A human monoclonal antibody blocking SARS-CoV-2 infection
Source: Nat Commun. 2020 May 4;11:2251. doi: 10.1038/s41467-020-16256-y (PMC7198537; doi:10.1038/s41467-020-16256-y)
Supplement: Supplementary file 2 — Reporting Summary [file 41467_2020_16256_MOESM2_ESM.pdf]

nature research

Corresponding author(s): Berend-Jan Bosch

Last updated by author(s): Apr 20, 2020

Reporting Summary

Nature Research wishes to improve the reproducibility of the work that we publish. This form provides structure for consistency and transparency in reporting. For further information on Nature Research policies, see Authors & Referees and the Editorial Policy Checklist.

Statistics

For all statistical analyses, confirm that the following items are present in the figure legend, table legend, main text, or Methods section.

n/a	Confirmed	
☐	☒	The exact sample size (*n*) for each experimental group/condition, given as a discrete number and unit of measurement
☐	☒	A statement on whether measurements were taken from distinct samples or whether the same sample was measured repeatedly
☒	☐	The statistical test(s) used AND whether they are one- or two-sided<br>*Only common tests should be described solely by name; describe more complex techniques in the Methods section.*
☒	☐	A description of all covariates tested
☒	☐	A description of any assumptions or corrections, such as tests of normality and adjustment for multiple comparisons
☐	☒	A full description of the statistical parameters including central tendency (e.g. means) or other basic estimates (e.g. regression coefficient) AND variation (e.g. standard deviation) or associated estimates of uncertainty (e.g. confidence intervals)
☒	☐	For null hypothesis testing, the test statistic (e.g. *F*, *t*, *r*) with confidence intervals, effect sizes, degrees of freedom and *P* value noted<br>*Give P values as exact values whenever suitable.*
☒	☐	For Bayesian analysis, information on the choice of priors and Markov chain Monte Carlo settings
☒	☐	For hierarchical and complex designs, identification of the appropriate level for tests and full reporting of outcomes
☒	☐	Estimates of effect sizes (e.g. Cohen's *d*, Pearson's *r*), indicating how they were calculated

Our web collection on statistics for biologists contains articles on many of the points above.

Software and code

Policy information about availability of computer code

Data collection	Online protein sequence alignment tool: https://www.ebi.ac.uk/Tools/msa/clustalo/
Data analysis	GraphPad Prism (version 8)<br>CytoFLEX Flow Cytometer (Beckman Coulter)<br>FlowJo (version 10)<br>Immunospot S6 ULTIMATE Analyzer(CTL)<br>Fortebio Data Analysis 7.0

For manuscripts utilizing custom algorithms or software that are central to the research but not yet described in published literature, software must be made available to editors/reviewers. We strongly encourage code deposition in a community repository (e.g. GitHub). See the Nature Research guidelines for submitting code & software for further information.

Data

Policy information about availability of data

All manuscripts must include a data availability statement. This statement should provide the following information, where applicable:

- Accession codes, unique identifiers, or web links for publicly available datasets
- A list of figures that have associated raw data
- A description of any restrictions on data availability

Figures that have associated raw data are: Fig.1 and 2, Suppl. Fig.2-6. The data that support the findings of this study are available from the corresponding author upon reasonable request.

Field-specific reporting

Please select the one below that is the best fit for your research. If you are not sure, read the appropriate sections before making your selection.

☒ Life sciences ☐ Behavioural & social sciences ☐ Ecological, evolutionary & environmental sciences

For a reference copy of the document with all sections, see nature.com/documents/nr-reporting-summary-flat.pdf

Life sciences study design

All studies must disclose on these points even when the disclosure is negative.

Sample size	For FACS-based experiments, a standard number of 10.000 cells/sample was analysed.<br>For infection experiments, 50,000 cells were taken per sample. Infection dose of authentic virus infection experiments was based on MOI to prevent double infections of cells, or on luciferase counts (for luciferase-based VSV pseudotyping exp.) to be in linear range of measurement.
Data exclusions	n.a.
Replication	All experiments were replicated successfully
Randomization	To allow accurate comparison between the data presented in the figures, the substrates that were used for each figure were grouped and the data was obtained at the same day. Randomization is not applicable in this case.
Blinding	Blinding was not relevant as the reported data was not based on subjective observations, but quantitative measurements, including FACS, ELISA etc.

Reporting for specific materials, systems and methods

We require information from authors about some types of materials, experimental systems and methods used in many studies. Here, indicate whether each material, system or method listed is relevant to your study. If you are not sure if a list item applies to your research, read the appropriate section before selecting a response.

Materials & experimental systems

n/a	Involved in the study	
☐	☒	Antibodies
☐	☒	Eukaryotic cell lines
☒	☐	Palaeontology
☒	☐	Animals and other organisms
☒	☐	Human research participants
☒	☐	Clinical data

Methods

n/a	Involved in the study	
☒	☐	ChIP-seq
☐	☒	Flow cytometry
☒	☐	MRI-based neuroimaging

Antibodies

Antibodies used	1. StrepMAB-Classic, HRP conjugate, Cat.no: 2-1509-001, IBA, 1:2,000<br>2. Goat anti-Human IgG (H+L) Cross-Adsorbed Secondary Antibody Alexa Fluor 594, Catalog # A-11014, Invitrogen, 1:200<br>3. Goat anti-Mouse IgG (H+L) Highly Cross-Adsorbed Secondary Antibody, Alexa Fluor 594, Catalog # A-11032, Invitrogen, 1:200<br>4. Anti-IgG2c Antibody (HRP) (rat), Catalog#KT99, Absea Biotechnology, 1:2,500<br>5. Anti-IgG2b Antibody (HRP) (rat), Catalog#KT98, Absea Biotechnology, 1:2,500<br>6. Anti-IgG1 Antibody (HRP) (rat), Catalog#KT96, Absea Biotechnology, 1:2,500
Validation	Primary antibodies have been validated by different assays including ELISA, IFA and FACS, as described in the manuscript. All the secondary antibodies were bought from commercial vendors and were validated by the manufacturers.

Eukaryotic cell lines

Policy information about cell lines

Cell line source(s)	1. African green monkey origin, Vero-E6, from ATCC<br>2. African green monkey origin, Vero-CCL81, from ATCC<br>3. Human embryonic kidney cell, HEK293T, from ATCC
Authentication	All the cells were derived from and authenticated by ATCC

Mycoplasma contamination	We confirm that all cells were tested as mycoplasma negative.
Commonly misidentified lines (See ICLAC register)	No commonly misidentified cell lines were used.

Flow Cytometry

Plots

Confirm that:

- [x] The axis labels state the marker and fluorochrome used (e.g. CD4-FITC).
- [x] The axis scales are clearly visible. Include numbers along axes only for bottom left plot of group (a 'group' is an analysis of identical markers).
- [x] All plots are contour plots with outliers or pseudocolor plots.
- [x] A numerical value for number of cells or percentage (with statistics) is provided.

Methodology

Sample preparation	HEK-293T cells were seeded at a density of 2.5×10E5 cells per ml in a T75 flask. After reaching 70~80% confluency, cells were transfected with an expression plasmid encoding human ACE2 - C-terminally fused to the GFP - using Lipofectamine 2000 (Invitrogen). Two days post transfection, cells were dissociated by cell dissociation solution (Sigma-aldrich, Merck KGaA; cat. no. C5914). Cells were then fixed by incubation with 2% paraformaldehyde in PBS for 20 min at room temperature and subject to antibody staining.
Instrument	Flow cytometry was performed using CytoFLEX LX Flow Cytometer, Beckman Coulter
Software	Data were acquired by CytExpert Software, then analyzed using FlowJo software V.10
Cell population abundance	N/A
Gating strategy	FSC/SSC gates were used to select mononuclear cells. Control antibody staining was used to define positive/negative cell populations.

- [x] Tick this box to confirm that a figure exemplifying the gating strategy is provided in the Supplementary Information.
